# Home-Based Individualized Cognitive Stimulation (iCS) Therapy in Portuguese Psychiatric Patients: A Randomized Controlled Trial

**DOI:** 10.3390/brainsci12121655

**Published:** 2022-12-02

**Authors:** Susana I. Justo-Henriques, Enrique Pérez-Sáez, Janessa O. Carvalho, Marco Cavallo, Paula Sargaço

**Affiliations:** 1 Health Sciences Research Unit: Nursing (UICISA: E), Nursing School of Coimbra (ESEnfC), 3004-011 Coimbra, Portugal; 2 National Reference Centre for Alzheimer’s and Dementia Care, Imserso, 37008 Salamanca, Spain; 3 Department of Psychology, Bridgewater State University, Bridgewater, MA 02325, USA; 4 Faculty of Psychology, eCampus University, 22060 Novedrate, CO, Italy; 5 Hospital Infante D. Pedro, Centro Hospitalar do Baixo Vouga, 3810-501 Aveiro, Portugal

**Keywords:** basic and instrumental activities of daily living, cognitive function, executive function, home-based interventions, individual cognitive stimulation, mood, psychotic disorder, quality of life

## Abstract

Cognitive difficulties are common in people with mental health issues, including psychotic disorders, although this population may have difficulty accessing treatments due to various challenges, including transportation, remembering appointments, or discomfort in crowded or unfamiliar places. Home-based services can be crucial and effective for reaching populations with accessibility issues; one home-based intervention technique is individual cognitive stimulation (iCS), which has been shown to be an effective strategy to target and improve cognitive functioning in various samples. Using a previously established Portuguese iCS protocol, based on an initial brief cognitive assessment and the subsequent administration of cognitive stimulation materials and reflection exercises, the current randomized controlled trial explored the effectiveness of the iCS intervention on participants in Portugal with psychotic disorders. Outcome tools included measures of cognition, depression, quality of life, and functional abilities at baseline, the completion of the intervention, and post-intervention follow-up. With two well-matched groups at baseline, the results revealed significant improvements in the intervention group on cognitive functioning, depression, quality of life, and, more modestly, functional activities. These results offer an important contribution to the field of iCS protocols, in an effort to enhance the lives and well-being of various clinical populations, including those with psychotic disorders.

## 1. Introduction

Mental health issues in Portugal are at a high level, with prevalence rates of depression affecting 5.7% of the population and 4.9% afflicted with anxiety [1]. In fact, epidemiological data revealed that Portugal has one of the highest rates of lifetime prevalence of mental illness in Europe [2]. Recently, it has been estimated that one in five Portuguese suffer from a psychiatric disorder (such as depression, anxiety, and psychotic disorders) [3]. The global health disaster related to coronavirus disease (i.e., COVID-19) and the pandemic has further increased the rates of mental health disorders, which is, in part, due to increased isolation, anxiety, reduced access to care, financial distress, and concerns about illness. Worry for the health of loved ones also had a significant impact on mental health, and the urgent need to take care of others affected everyday life. As seen in the elevated rates worldwide, residents of Portugal also have experienced significantly increased rates of mental health disorders relative to the figures prior to the COVID-19 pandemic [4].

Treatment interventions for mental health disorders are limited in both number and accessibility. Portugal’s government offers its citizens the *Cuidados Continuados Integrados* (Integrated Continued Care), a mental health care network; however, this program is limited and also small in scope, which is a major constraint for providing a broad spectrum of rehabilitation and support services for persons with mental health problems [5]. The *Programa Nacional para a Saúde Mental* (National Mental Health Program 3]) has also sought to provide mental health programs to Portuguese citizens; in this program, most home-support intervention teams include nurses and social workers, although, regrettably, psychologist and neuropsychologist involvement is limited in these programs despite the fact that psychologists are ideally suited to provide comprehensive interventions for mental health disorders and other consequences of chronic mental health conditions, such as cognitive decline [6].

The effects of mental health disorders are pervasive and debilitating and often include cognitive deficits, both acutely, during a psychiatric episode, and chronically. There are well-established impairments in cognitive functioning in persons with depression [7], anxiety [8], and, within the topic of the current study, psychotic disorders [9,10]. Cognitive deficits in persons with psychotic disorders include executive functioning [11,12], memory [11,12,13], and attention [14]. The effects of these deficits are broad; in a sample of psychiatric patients, executive functioning mediated their global real-world functioning [15].

The need to focus on cognitive interventions in those with mental health disorders is implicated, as long-term cognitive aging in some domains may be accelerated in persons with psychotic disorders [16]. Specifically, patients who were admitted for inpatient psychiatric hospitalization showed greater cognitive impairments relative to their same-aged peers in a 20-year follow-up. However, while past research is limited, cognitive interventions can be helpful in psychiatric patients to improve cognitive and social dysfunction. For example, a computerized on-site cognitive intervention in a small sample of Italian patients with psychotic disorders has demonstrated positive effects in cognitive functions and social cognition [17]. These results emphasize the importance of interventions focused on cognitive functioning in patients with psychosis, even after acute psychotic symptoms have improved.

Home-based interventions offer many benefits and conveniences to patients, including those with significant mental illness. Patients who may especially benefit include those with limited transportation access, those who have trouble remembering scheduled appointments, those uncomfortable in crowded environments, and those with concerns about presenting themselves at an unfamiliar location.

Home-based interventions have been successful in situations of significant *medical* illnesses. For example, in Portugal, the *Samaritano* project, a home-based intervention for participants with terminal illnesses, led by a team of nurses and psychiatrists, produced significant improvements in reported quality of life [18]. However, few published studies have explored the benefits of home-based intervention in persons with chronic *mental* illnesses. Borson et al. [19] conducted home-based psychotherapy sessions in older adults and adult caregivers for the treatment of depression and anxiety. Werbeloff et al. [20] had success with crisis intervention teams, including in non-affective psychosis patients with one-time interventions in certain situations. In Germany, Sakellaridou et al. [21] developed assertive community and at-home interventions over a 24-month period in persons affected by psychosis, leading to a significant improvement in quality of life, as well as a reduction in hospital admissions. Landers et al. [22] suggested viable options for activating home-based health care in the US, focusing mainly on medical and nursing services. A further challenge regarding access to care in all populations is seen in the restrictions implemented as a result of the COVID-19 pandemic, emphasizing the need for the continued development of empirically supported interventions.

With known cognitive deficits in psychotic disorders and the need for providing accessible care to persons with these disorders, the benefits of developing successful evidence-based at-home cognitive interventions are numerous and broad. Cognitive stimulation (CS) is an intervention that seeks to improve cognitive functions, social functioning, and QoL by providing an enriching and engaging environment [23,24]. A previous randomized controlled trial using cognitive therapy on patients with psychotic disorders in the United States was successful at improving not only attention and memory but also functional abilities and quality of life [25]. In this case, the intervention took place at a clinic, and dissemination was completed in a group format.

A previously established Portuguese individual cognitive stimulation (iCS) protocol [26,27] utilized reminiscence (which involved discussing past significant experiences with participants) and sought to target cognition by discussing past events and providing cues from the past, such as photographs, objects, or music [28]. Reminiscence-based interventions can be individualized and may seem less distressing and more engaging to participants while promoting communication and enabling participants to connect with their past and regain their sense of personal identity [29,30]. A recent randomized clinical trial (RCT), using an iCS protocol that is detailed elsewhere [31], found significant positive effects on cognitive domains and QoL in a Portuguese mixed neurocognitive disorder sample after 13 weeks, compared to those who did not receive the iCS protocol [26,27]. A study piloting this home-based individualized cognitive intervention in Portuguese adults with psychotic disorders revealed significant positive effects from the intervention regarding global cognition, executive function, and depressive symptoms [32].

The current study sought to build upon these previous findings by exploring the effects of the home-based iCS protocol on cognitive functioning, the activities of daily living, mood, and quality of life in a sample of Portuguese adults with psychotic disorders. In keeping with previous studies using different clinical targets (such as patients affected by dementia), we expected to find a significant treatment response mainly in the realm of cognitive functions (i.e., the specific target of our intervention), with a more modest impact on other psychological measures (such as the level of depression, quality of life, and functional measures) not specifically addressed by our intervention.

## 2. Materials and Methods

This study was registered on the ClinicalTrials.gov register (identifier: NCT04783285). The design is a single-blind, randomized, parallel two-arm (iCS vs. treatment as usual, 1:1 ratio), controlled clinical trial. After recruitment and baseline psychological assessments, the participants were randomly assigned to either the treatment group (receiving two 45-min weekly sessions of iCS for 16 weeks, in addition to their regular treatment) or the control group (receiving treatment as usual for 16 weeks). Assessment measures were administered at baseline (T0), after the end of the CS program (primary endpoint T1), and at 8-week follow-up (T2).

### 2.1. Participants

Participants were randomly allocated to either the intervention or the control group on a 1:1 ratio. A non-stratified permuted block randomization process (with variable block sizes) was carried out using the software RandList (DatInf GmbH, Tübingen, Germany) with no knowledge of participants’ baseline scores and demographic variables. Participants, therapists, and institution staff remained blinded to the participant groups until the intervention started. Allocation to groups was concealed from the evaluator until follow-up assessments were completed. The researcher who communicated with the institution assessed the participants’ eligibility and enrolled them in the study. The therapist at the institution assigned the participants to groups.

**Figure 1:**
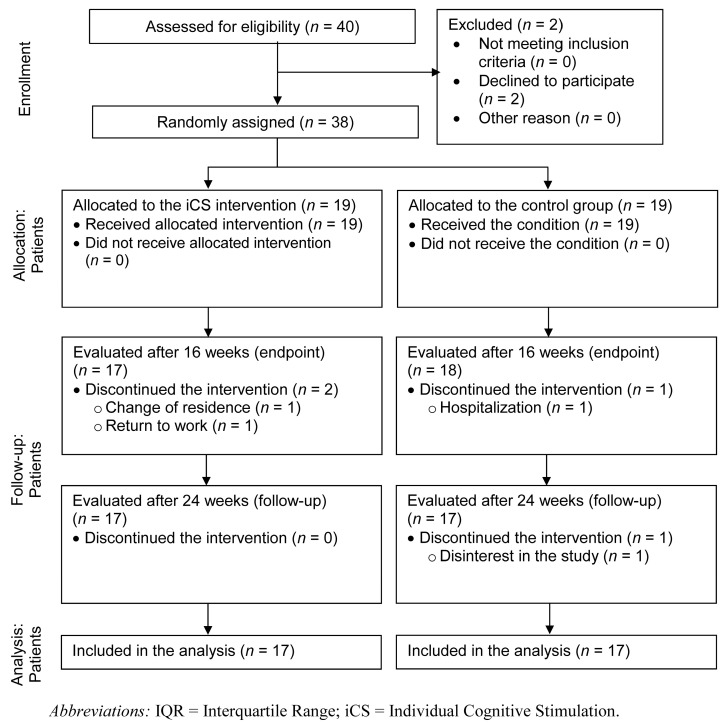
CONSORT diagram of participant flow through the study.

Participants were diagnosed with psychotic disorders, according to the Diagnostic and Statistical Manual of Mental Disorders (DSM-5) [33] criteria, from a cohort of patients who presented at the psychiatry service of the Hospital of Aveiro (residents of the municipality of Albergaria-a-Velha). After the baseline assessment, 38 participants were selected: 19 participants were assigned to the intervention group, and 19 to the control group (see Figure 1).

The inclusion criteria for the study were as follows: (a) adults under 65 years; (b) diagnosed with being on the schizophrenia spectrum and other psychotic disorders, according to the criteria of DSM-5 [33], as determined by a clinician; (c) willing to participate in all intervention and assessment sessions; (d) providing informed consent; (e) native Portuguese speakers. The exclusion criteria were: (a) presenting with a condition requiring immediate intervention (e.g., suicidal thoughts); (b) severe sensory and physical limitations that prevent participation and engagement; (c) severe delusions/disconnection with the environment and very limited attention; (d) inability to communicate adequately; (e) psychoactive substance use; (f) currently participating in another study.

More participants were women (53%), single (55%), and middle-aged (*M* = 53 years old). Participants had an average of around 4 years of education. Regarding their living situation, 32% lived alone, while others cohabitated (e.g., spouse, family, or unmarried partners). Only 21% of participants received home support services. The most common clinical diagnosis was schizophrenia (Table 1).

Prior to obtaining informed consent from all subjects or their legal representatives, written information was provided to all interested parties, which included the research protocol (duration, nature, and number of sessions), data-handling according to the rules of the privacy and protection protocols of patient information, reminders that participation is voluntary, and the fact that participants can withdraw consent and participation without any negative effects on the care and treatment received from their institution.

All the administered procedures met the ethical standards of the institutional committee on human experimentation and aligned with the Helsinki Declaration. The Health Sciences Research Unit: Nursing of Coimbra approved the project (approval number 752/02-2021).

### 2.2. Intervention

The home-based individualized intervention took place between April and October 2021 and consisted of 32 sessions, each lasting 45 min. The iCS protocol used has been described elsewhere, including specific activities and materials for each session [32]. All sessions followed the same structure, based on the model implemented by Justo-Henriques et al. [34,35,36,37], including providing complementary materials for the main activity. The first minutes of each session were aimed at introducing the work to be done, with the subsequent minutes focused on reality orientation: here, the patient was encouraged to complete information regarding the current date, time, and geographical location. Then, the core cognitive stimulation materials were related to various cognitive domains, including attention, memory, language, executive functions, and calculations. Each session followed a specific theme (e.g., Portuguese music, family tree, well-known Portuguese actors, sequencing time of day, or a grocery-shopping simulation exercise). The end of each session was dedicated to a brief relaxation exercise and a short discussion of the possible difficulties and the pros and cons of the session just ended. After the conclusion of the session, the therapist completed an evaluation. For a detailed presentation of the content of each session, see Tables S1 and S2 in the Supplementary Materials. The participants in the control group did not receive the iCS but received their usual treatment as provided by the Institution, which encompassed scheduled socialization and regular group participation, as elected. Assessments were completed at the same intervals as those scheduled for the treatment group.

### 2.3. Instruments

Participants were assessed by a trained evaluator blinded to the participant group at three time-points: a baseline assessment before the intervention (T0), an endpoint assessment at 16 weeks after the baseline (T1), and a follow-up assessment at 24 weeks after the baseline (T2). The primary outcome measure was cognitive function, with secondary outcome measures of the basic and instrumental activities of daily living, mood, and quality of life. The Portuguese version of each assessment instrument was used.

Cognitive functioning was assessed using the Montreal cognitive assessment (MoCA) model [38,39], version 7.1, which explores eight domains: visuospatial/executive, naming, memory, attention, language, abstraction, delayed recall, and orientation. This measure boasts strong test/retest reliability (Cronbach’s alpha = 0.90). Scores range from 0–30 points, higher scores indicate better cognitive functioning.

Executive functions were evaluated by using the frontal assessment battery (FAB) [40,41], which takes into account six different sub-cognitive domains: abstract thinking, mental flexibility, motor programming, interference sensibility, inhibitory control, and environmental independence. Scores range from 0–18 points, with higher scores indicating better performance.

The activities necessary for daily life were evaluated using the instrumental activities of daily living scale (IADL) [42,43]. This is an 8-item measure of a participant’s ability to complete ADLs (e.g., telephone use, doing laundry, preparing food, handling finances, and making purchases). Scores ranged from 8 to 30 points, with higher scores indicating greater functional difficulties.

The Center for Epidemiologic Studies depression scale (CES-D) [44,45] was used to assess each participant’s mood. It is a 20-item self-reported measure in which participants rate how often over the past week they experienced symptoms associated with depression (e.g., restless sleep, poor appetite, and feeling lonely). Higher scores indicate more severe depressive symptoms.

Quality of life was evaluated through the World Health Organization’s quality of life measure (WHOQOL-Bref) [46,47], an instrument that assesses the participant’s perception of their quality of life within the context of their culture and values, along with their goals, standards, and concerns. It comprises 26 items evaluating the perception of physical health, psychological health, social relationships, environment, and general health. The global score is scored from 0 to 100, with higher scores reflecting a better self-reported quality of life.

Quality of life also was evaluated with the 36-item MOS short-form health survey, volume 2 (SF-36v2) [48,49,50]. Consisting of 36 items, it assesses eight dimensions of health (physical functioning, role limitations due to physical health or emotional problems, intensity and discomfort due to pain, general health, vitality (i.e., energy/fatigue), social functioning, and emotional well-being). The global score ranges from 0 to 100, with higher scores indicating a better health-related quality of life.

### 2.4. Data Analysis

Chi-square tests for the categorical variables and *t*-tests for the continuous variables were conducted to explore group variability. No imputation of missing data was made; thus, only data from participants who completed the follow-up assessment were analyzed.

The effects of iCS on outcomes (MoCA, FAB, IADL, CES-D, WHOQOL-Bref, and SF-36v2) were analyzed using a 2 × 3 repeated-measures mixed ANOVA, with group assignments as the between-subjects factor (iCS and control) and time as the within-subjects factor (baseline T0, endpoint T1, and follow-up, T2). The main effects of interest were the group x time interactions. The Greenhouse–Geisser correction was used when the sphericity test was significant. Pairwise comparisons (Bonferroni correction) were also used to analyze the differences between time points and between groups. The level of significance was set at *p* < 0.05 for all analyses. Statistical analysis was performed using IBM SPSS Statistics, version 20.

## 3. Results

### 3.1. Sociodemographic and Clinical Characteristics

Recruitment took place from April to October 2021. A total of 38 participants were selected: 19 (50%) were allocated to the iCS group and 19 (50%) were allocated to the control group. As can be seen from Table 1, no significant differences were found between groups regarding the demographic factors considered (e.g., age, gender, clinical diagnosis, level of education, marital status, frequency of institution attended, and residential situation). No significant differences were found between the intervention and control groups regarding clinical condition or baseline mean scores in the psychological measures administered (e.g., MoCA, FAB, IADL, CES-D, WHOQOL-Bref, and SF-36v2). Further details are in Table S3 of the Supplementary Materials.

Of the 19 participants in the iCS group, 17 (89.5%) completed the three assessments. The intervention could not be completed for 2 participants (10.5%) who were not assessed at T1 (see Figure 1). One of the participants withdrew from the study due to a change of residence and another participant withdrew due to returning to work. No serious adverse events related to the trial were recorded.

Of the 19 participants in the control group, one (5.3%) did not complete the endpoint assessment due to hospitalization, while another patient (5.3%) could not be assessed at follow-up due to uninterest in the study, resulting in 17 participants (89.5%) completing the three assessments.

At the follow-up assessment, no significant differences were found between the intervention and control groups regarding demographic variables, clinical condition, or baseline mean scores, the only exception being the physical functioning domain of the SF-36v2 (*p* = 0.049).

### 3.2. Effects of iCS at the Endpoint and Follow-Up

#### 3.2.1. MoCA

The 2 × 3 ANOVA (Table 2) showed a significant group x time interaction, F(1.640, 52.475) = 26.672, *p* < 0.001, *η_p_*^2^ = 0.455. At the endpoint, pairwise comparisons showed that the iCS group significantly improved on MoCA scores (*p* < 0.001), and this improvement was maintained at follow-up (*p* < 0.001). The control group scores stayed consistent throughout the trial (*p* = 0.645 at T1, *p* = 0.253 at T2). The iCS and control groups significantly differed at the endpoint (*p* = 0.002) and follow-up (*p* = 0.001).

#### 3.2.2. FAB

The ANOVA for the FAB scores (Table 2) showed a significant Group x Time interaction, where *F* (2, 64) = 16.122, *p* < 0.001, and *η_p_*^2^ = 0.335. The iCS group significantly improved their FAB scores at the endpoint (*p* < 0.001), maintaining it at follow-up (*p* < 0.001). The control group maintained their scores over the course of the study (*p* = 0.839 at T1, *p* = 0.226 at T2). The iCS and control groups significantly differed at the endpoint (*p* = 0.032) and follow-up (*p* = 0.009).

#### 3.2.3. IADL

The ANOVA (Table 2) showed a significant group x time interaction, where *F* (1.506, 48.192) = 13.599, *p* < 0.001, and *η_p_*^2^ = 0.298. Pairwise comparisons showed that the iCS group maintained their IADL scores over the course of the study (*p* = 0.086 at T1, *p* = 0.085 at T2). The control group worsened their scores over the course of the study, with significant differences at follow-up when comparing both to the baseline (*p* = 0.003) and the endpoint (*p* = 0.028). However, the iCS and control groups did not significantly differ at any time point (*p* = 1.000 at T1, *p* = 0.683 at T2).

#### 3.2.4. CES-D

The ANOVA for CES-D (Table 2) did not show a significant group x time interaction *F* (1.291, 41.324) = 1.869, *p* = 0.177, *η_p_*^2^ = 0.055, since both iCS and control groups maintained their scores throughout the assessments. However, the iCS and control group significantly differed at the endpoint (*p* = 0.004) and follow-up (*p* = 0.001), with lower scores for the iCS group.

#### 3.2.5. WHOQOL-Bref

The ANOVA for the WHOQOL-Bref domains (Table 3) showed significant group × time interactions for *physical health*, *social relationships*, *environment,* and *in general*. For the iCS group, the *psychological health*, *social relationships*, and *environment* domain scores improved at the endpoint and the improvements were maintained at follow-up. The control group maintained its scores in all domains over the course of the study. The iCS and control groups significantly differed at the endpoints and follow-up points for the *psychological health*, *social relationships*, *environment*, and *general* domains.

#### 3.2.6. SF-36v2

The ANOVA for the SF-36v2 domains (Table 4) showed significant group × time interactions for the domains of *physical functioning*, *role limitations (physical)*, *bodily pain*, *role limitations (emotional)*, *mental health,* and *energy/vitality*. For the iCS group, the *physical functioning*, *role limitations (physical)*, and *role limitations (emotional)* domain scores improved after the intervention. The improvements in *role limitations (physical)* and *role limitations (emotional)* were maintained at follow-up, while the scores regressed for *physical functioning.* The control group maintained their scores throughout the study, except for significant decreases in *role limitations (physical)* and *bodily pain*. The iCS and control groups significantly differed at the endpoint and follow-up for all domains except for *physical functioning.*

## 4. Discussion

Over the last decade, home-based health interventions have gradually been proven to be effective and accessible for patients, especially those with significant mental illness who may have limited transportation access, trouble remembering scheduled appointments, and concerns about presenting to an unfamiliar location. Since the COVID-19 pandemic, the need to develop and improve this type of intervention has become universally evident. Interesting points pertain not only to the possible association between COVID-19 infection and long-term cognitive deficits but also to the general issue of a potential relationship between the fear of COVID-19 and the alteration of specific cognitive abilities. For example, it could be useful to investigate how the fear of COVID-19 infection, which has been reported as a major psychological stressor during the disease outbreak, is associated with a fear response and can have an impact on cognitive abilities, including executive functions such as action control [51], rapid vigilance, and episodic memory [52,53].

CS interventions that target patients affected by psychiatric disorders seek to improve their cognitive abilities, social functioning, and, ultimately, their QoL by providing an enriching and engaging environment. Our research group developed an iCS protocol, used in a recent RCT, that showed a significant positive effect of the iCS on cognitive screening, memory, and QoL in a Portuguese mixed neurocognitive disorder sample [26,27]. The adaptation of this protocol into a home-based iCS intervention was promising in patients with psychotic disorders [32].

Here, we investigated the feasibility and efficacy of the above-mentioned home-based iCS intervention on adults with psychotic disorders. The two groups of participants that were recruited were well-matched in terms of age, gender, level of education, and marital and residential status. Additionally, the two groups did not differ significantly on baseline clinical and cognitive assessments. The cognitive outcomes showed a significant difference in the two groups, with the experimental group reporting higher scores (reflecting better performance) with certain measures (i.e., MoCA and FAB). Similarly, the iCS had a notable positive impact on the presence of depressive symptoms (as measured by the CES-D), while its impact on IADLs was less notable (although there was a decline in the control group across the time points). This may be explained by considering the nature of both the iCS and the administered tools: while a cognitive stimulation program is expected to impact specific cognitive measures, improved IADLs (e.g., shopping, cooking, or managing finances) depends not only on well-defined cognitive abilities but also on general motor skills, the characteristics of the environment, the availability of social supports, etc. As a result, our RCT significantly impacted the cognitive measures and (more modestly) the functional measures. One more controversial conclusion is the interpretation of the impact on depressive symptoms. Possibly due to seeing their cognitive performance gradually improving over time, patients experienced an increase in their self-esteem, reducing their depressive symptoms. Alternatively, a significant involvement in an active process of cognitive stimulation could be via fueling one’s self-efficacy, thus leading to the participants experiencing fewer neuropsychiatric symptoms. However, this is purely theoretical speculation; thus, further studies should specifically address this interesting topic.

Quality of life was assessed at all time points, using two measures (WHOQOL-Bref and SF-36 (version 2), as we believe that any psychological intervention should have a *general* positive impact on the patients’ QoL, beyond the *specific* effect expected from each planned intervention (i.e., stimulating cognitive performance or limiting the presence of depressive symptoms, etc.). Interestingly, our results showed that in the experimental group, each QoL dimension improved from the beginning of the intervention, with little change in the control group. We also demonstrated that the improvements in these QoL dimensions were significantly higher in the experimental group. Previous studies targeting neuropsychiatric patients and healthy older adults have already reported the association between cognitive interventions and QoL improvement (e.g., Grasso et al. [54]; Lok et al., [55]; Kazazi et al. [56]), whereas others failed to find such an association (e.g., Orrell et al. [57]). Potentially, since the QoL measures used included an assessment of cognition, the intervention modified our QoL results as well. Alternatively, we may assume that the patients’ achievements during the interventions increased their self-esteem and self-efficacy, in turn, positively impacting their QoL.

This study has various strengths. First, random group allocation and similarly matched baseline clinical and socio-demographic variables supported the two groups as being homogeneous and highly comparable. Second, a detailed assessment encompassing numerous cognitive, functional, and QoL measures was conducted at multiple time points. While various studies have included briefer cognitive and psychological assessments, our evaluation was broader. Finally, the follow-up allowed us to investigate the stability of the results over time. The vast majority of participants completed the two-month follow-up (34/38, or >89%), and we showed that the patients in the iCS group maintained an improvement on most of the assessment measures.

This study also has some limitations. First, the sample size was relatively small (although consistent with typical RCT size), so future studies should aim to replicate these results with larger samples. Second, the regularly scheduled activities completed in the control group were conducted at the institution and not at patients’ residences, thus confounding whether the different setting (at home vs. in the clinic) may have played a role in this study. Lastly, a longer follow-up period could have provided further insight into the length of treatment effects.

## 5. Conclusions

The iCS interventions under study should be considered a viable, effective, and therapeutic option for psychiatric patients. Further studies should continue to explore intervention parameters (e.g., number of sessions weekly, session duration, treatment length) that may provide potentially greater and long-lasting beneficial cognitive outcomes. Defining the standardized and shared protocols of cognitive stimulation interventions, differentiated according to the type or stage (prodromal, initial, or advanced) of psychiatric diseases may be useful. Additionally, future studies should explore the factors affecting the long-term treatment effects. Finally, further research should identify the amount and nature of learning that patients affected by psychiatric conditions can reasonably achieve, and the extent of the generalizability of their newly acquired skills to everyday life tasks. For example, cognitive rehabilitation training, including the teaching of compensatory strategies and a discussion of the generalizability of cognitive skills to real life, offers a more significant impact on patients’ lives.

To conclude, the present RCT suggests that it is possible to stimulate at least some cognitive abilities successfully in patients affected by psychotic disorders. Our pattern of results showed that the treated patients were able to improve their performance on certain measures due to the cognitive intervention that was realized, and, above all, that these improvements were maintained over time for at least some weeks. However, although our study provided some relevant suggestions about this important clinical issue, more robust evidence is urgently needed to answer the essential question of what kind of cognitive training may play a significant role in fostering patients’ *general* cognitive and social abilities, beyond the impact demonstrated on the *specific* skills targeted by the intervention itself.

## Figures and Tables

**Table 1:** Sociodemographic and clinical characteristics of the sample, and the results of the between-group comparisons.

	Overall Sample (*n* = 38)	iCS Group (*n* = 19)	Control Group (*n* = 19)		
**Age**				*t*	*p*-value
Mean (SD)	52.87 (9.03)	52.63 (9.49)	53.11 (8.79)	−0.160	0.874
Range	34–64	36–64	34–64		
**Gender**				X^2^	*p*-value
Male	18 (47.4%)	8 (42.1%)	10 (52.6%)	0.422	0.746
Female	20 (52.6%)	11 (57.9%)	9 (47.4%)		
**Educational level**					
3 to 4 years	16 (42.1%)	7 (36.8%)	9 (47.4%)	4.568	0.206
5 to 6 years	8 (21.1%)	5 (26.4%)	3 (15.8%)		
7 to 11 years	11 (28.9%)	7 (36.8%)	4 (21.0%)		
Over 11 years	3 (7.9%)	0 (0%)	3 (15.8%)		
**Marital status**					
Single	21 (55.3%)	10 (52.6%)	11 (57.9%)	5.238	0.155
Married	7 (18.4%)	6 (31.6%)	1 (5.3%)		
Divorced	7 (18.4%)	2 (10.5%)	5 (26.3%)		
Widowed	3 (7.9%)	1 (5.3%)	2 (10.5%)		
**Home support service**					
No	30 (78.9%)	15 (78.9%)	15 (78.9%)	0.000	1.000
Yes	8 (21.1%)	4 (21.1%)	4 (21.1%)		
**Residential situation**					
Spouse	5 (13.2%)	4 (21.1%)	1 (5.3%)	3.133	0.372
Family	20 (52.6%)	10 (52.6%)	10 (52.6%)		

**Table 2:** Results of the repeated-measures ANOVA for MoCA, FAB, IADL, and CES-D scores. The results of the pairwise comparisons (Bonferroni correction) are given for iCS vs. the control group at baseline (T0), endpoint (T1), and follow-up (T2) assessments, as well as the results of the pairwise comparisons (Bonferroni correction) for iCS and control groups (T0 vs. T1, T0 vs. T2, and T1 vs. T2).

	iCS (*n* = 17)			Control (*n* = 17)			Moment #xD7; Group				Pairwise Comparisons								
	T0 Mean (SD)	T1 Mean (SD)	T2 Mean (SD)	T0 Mean (SD)	T1 Mean (SD)	T2 Mean (SD)	df	*F*	*p*	*η_p_* ^2^	iCS			Control			iCS vs. Control		
											T0 vs. T1	T0 vs. T2	T1 vs. T2	T0 vs. T1	T0 vs. T2	T1 vs. T2	T0	T1	T2
**MoCA**	18.82 (2.70)	22.12 (2.89)	22.06 (2.79)	18.35 (3.48)	17.76 (4.35)	17.41 (4.66)	1.640, 52.475	26.672	<0.001	0.455	<0.001	<0.001	1.000	0.645	0.253	0.920	0.662	0.002	0.001
**FAB**	10.24 (2.17)	11.82 (2.32)	11.94 (1.75)	10.41 (2.06)	10.06 (2.28)	9.82 (2.60)	2, 64	16.122	<0.001	0.335	<0.001	<0.001	1.000	0.839	0.226	1.000	0.809	0.032	0.009
**IADL**	15.94 (5.09)	15.29 (4.90)	15.12 (4.92)	14.59 (5.60)	15.29 (5.91)	15.88 (5.86)	1.506, 48.192	13.599	<0.001	0.298	0.086	0.085	1.000	0.053	0.003	0.028	0.467	1.000	0.683
**CES-D**	28.18 (10.17)	25.94 (7.59)	24.06 (6.69)	32.94 (12.54)	35.00 (9.15)	33.18 (8.36)	1.291, 41.324	1.869	0.177	0.055	0.914	0.187	0.154	1.000	1.000	0.176	0.233	0.004	0.001

*Abbreviations:* CES-D = the Center for Epidemiologic Studies depression scale; FAB = frontal assessment battery; IADL = Lawton instrumental activities of daily living scale; iCS = individual cognitive stimulation; MoCA = Montreal cognitive assessment; T0 = baseline assessment; T1 = endpoint assessment; T2 = follow-up assessment.

**Table 3:** Results of the repeated-measures ANOVA for the WHOQOL-Bref domain scores. The results of the pairwise comparisons (Bonferroni correction) are given for iCS vs. the control group at baseline (T0), endpoint (T1), and follow-up (T2) assessments, as well as the results of the pairwise comparisons (Bonferroni correction) for iCS and control groups (T0 vs. T1, T0 vs. T2, and T1 vs. T2).

	iCS (*n* = 17)			Control (*n* = 17)			Moment × Group				Pairwise Comparisons								
	T0 Mean (SD)	T1 Mean (SD)	T2 Mean (SD)	T0 Mean (SD)	T1 Mean (SD)	T2 Mean (SD)	df	*F*	*p*	*η_p_* ^2^	iCS			Control			iCS vs. Control		
											T0 vs. T1	T0 vs. T2	T1 vs. T2	T0 vs. T1	T0 vs. T2	T1 vs. T2	T0	T1	T2
**Physical Health**	46.64 (20.50)	53.78 (17.01)	51.47 (18.17)	48.53 (22.59)	43.49 (19.16)	40.97 (17.68)	1.237, 39.578	4.727	0.029	0.129	0.108	0.786	0.659	0.396	0.250	0.545	0.800	0.107	0.097
**Psychological Health**	45.59 (20.96)	58.82 (15.79)	59.56 (13.40)	41.67 (24.61)	40.93 (16.09)	43.63 (15.10)	1.139, 36.458	3.871	0.052	0.108	0.018	0.017	1.000	1.000	1.000	0.195	0.620	0.003	0.003
**Social relationships**	36.76 (21.86)	55.88 (20.57)	60.78 (15.24)	34.31 (23.18)	31.37 (22.92)	32.35 (20.17)	1.319, 42.207	10.570	0.001	0.298	0.002	<0.001	0.126	1.000	1.000	1.000	0.753	0.003	<0.001
**Environment**	42.18 (14.07)	55.51 (7.37)	53.13 (6.99)	46.88 (16.50)	39.52 (11.26)	38.42 (9.62)	1.192, 38.134	14.972	<0.001	0.319	0.002	0.012	0.203	0.145	0.072	1.000	0.370	<0.001	<0.001
**General**	56.62 (22.15)	68.38 (20.31)	66.91 (18.19)	55.15 (20.28)	50.00 (22.10)	50.74 (23.99)	1.220, 39.029	4.848	0.027	0.132	0.084	0.113	1.000	0.965	1.000	1.000	0.841	0.017	0.034

*Abbreviations:* T0 = baseline assessment; T1 = endpoint assessment; T2 = follow-up assessment; WHOQOL-Bref = World Health Organization Quality of Life-Bref.

**Table 4:** Results of the repeated-measures ANOVA for the SF-36v2 domain scores. Results of pairwise comparisons (Bonferroni correction) for iCS vs. the control group at baseline (T0), endpoint (T1), and follow-up (T2) assessments. The results of the pairwise comparisons (Bonferroni correction) for iCS and control groups are also given (T0 vs. T1, T0 vs. T2, and T1 vs. T2).

	iCS (*n* = 17)			Control (*n* = 17)			Moment × Group				Pairwise Comparisons								
	T0 Mean (SD)	T1 Mean (SD)	T2 Mean (SD)	T0 Mean (SD)	T1 Mean (SD)	T2 Mean (SD)	df	*F*	*p*	*η_p_* ^2^	iCS			Control			iCS vs. Control		
											T0 vs. T1	T0 vs. T2	T1 vs. T2	T0 vs. T1	T0 vs. T2	T1 vs. T2	T0	T1	T2
**General health**	37.88 (19.21)	44.06 (21.55)	43.41 (19.57)	30.76 (24.75)	26.82 (17.09)	28.18 (17.55)	2, 64	1.606	0.209	0.048	0.485	0.760	1.000	1.000	1.000	1.000	0.356	0.015	0.023
**Physical functioning**	56.47 (35.34)	68.53 (28.98)	53.53 (30.25)	78.24 (25.86)	68.24 (26.34)	66.76 (25.37)	2, 64	4.880	0.011	0.132	0.041	1.000	0.010	0.114	0.156	1.000	0.049	0.975	0.176
**Role limitations (physical)**	51.10 (26.54)	67.65 (12.74)	68.38 (15.85)	67.65 (23.72)	48.53 (18.16)	52.94 (17.65)	1.396, 44.664	12.706	<0.001	0.284	0.036	0.035	1.000	0.013	0.089	0.545	0.064	0.001	0.009
**Bodily pain**	49.29 (27.01)	56.00 (20.47)	56.29 (17.55)	58.94 (28.07)	41.12 (16.67)	43.00 (17.59)	1.579, 50.533	6.154	0.007	0.161	0.865	0.803	1.000	0.022	0.045	1.000	0.315	0.027	0.035
**Role limitations (emotional)**	47.06 (24.82)	70.59 (11.83)	70.59 (13.54)	60.78 (25.98)	51.96 (15.74)	58.33 (12.84)	1.306, 41.784	9.882	0.001	0.236	0.002	0.002	1.000	0.526	1.000	0.095	0.125	0.000	0.011
**Social functioning**	50.00 (26.88)	63.97 (22.48)	65.44 (17.42)	40.44 (25.59)	38.24 (19.50)	41.91 (15.90)	1.523, 48.745	2.418	0.112	0.070	0.120	0.056	1.000	1.000	1.000	1.000	0.296	0.001	<0.001
**Mental health**	42.94 (21.22)	52.94 (21.73)	58.82 (19.08)	35.59 (24.99)	26.18 (18.84)	32.94 (16.30)	1.579, 50.542	4.058	0.032	0.113	0.399	0.023	0.444	0.470	1.000	0.293	0.362	0.001	<0.001
**Energy/vitality**	31.18 (15.67)	37.5 (14.27)	41.47 (18.60)	29.71 (16.63)	25.00 (15.10)	27.94 (12.38)	2, 64	3.731	0.029	0.104	0.274	0.042	0.411	0.583	1.000	0.852	0.792	0.020	0.018

*Abbreviations:* SF-36v2 = MOS Short-Form Health Survey (36 Items) v2; T0 = Baseline assessment; T1 = Endpoint assessment; T2 = Follow-up assessment.

## Data Availability

Not applicable.

## Supplementary Materials

The following supporting information can be downloaded at: https://www.mdpi.com/article/10.3390/brainsci12121655/s1, Table S1: Basic structure of the cognitive stimulation program in individual format; Table S2: contents of the main activities of the individual cognitive stimulation program; Table S3: Baseline scores of the outcomes. Results of between-group comparisons at baseline.
