# Supplementary material for: Home-Based Individualized Cognitive Stimulation (iCS) Therapy in Portuguese Psychiatric Patients: A Randomized Controlled Trial
Source: Brain Sci. 2022 Dec 2;12(12):1655. doi: 10.3390/brainsci12121655 (PMC9775072; doi:10.3390/brainsci12121655)
Supplement: Supplementary file 1 [file brainsci-12-01655-s001.zip › brainsci-2065025-supplementary.pdf]

Table S1

Basic Structure of The Cognitive Stimulation Program in Individual Format

Duration	Contentes	Activities
15 minutes	Beginning of session	Greeting<br>Mood check<br>Communicate the objectives of the session
	Reality orientation	Using a time orientation chart, identify the temporal and spatial elements of the day
25 minutes	Cognitive stimulation	Using therapeutic materials (e.g., software *RehaCom*©, *Livro do Presente e do Passado*© [Book of the Past and the Present], *Roletas da Memória*© [Memory Roulettes]), images, cards, stories and sounds. The following cognitive domains were trained: attention, language, short term memory, declarative memory, sensory memory, calculation, executive function, gnosis and praxis.
5 minutes	End of session	Analysis of the difficulties, interests, and benefits of the session<br>Return to calm<br>Farewell<br>Evaluation of the session in a specially designed register

Table S2

Contents of the Main Activities of the Individual Cognitive Stimulation Program

Session	Contents and intervention activities	Material	Stimulated cognitive domain
1	Pre evaluation (baseline)	n/a	n/a
2 and 19	*Activity(ies):* Place cards with incomplete words on each of the eight parts that form the roulette wheel. Give the participant individual cards with the letters that compose the alphabet; ask them to select and place the cards with the missing letters; evoke synonymous or associated words; sort alphabetically; memorize words; develop a theme.	Roulette and cards, on paper, with incomplete letters and words related to the theme "Portuguese language" (e.g. RM).	Attention, language, and memory.
3 and 20	*Activity(ies):* Place the cards with various pictures on the table (e.g. sound bingo I). Then ask the participant to identify the card with the picture corresponding to the sound. Play back the sounds from the pictures, identify the missing sounds, group the cards by topic (e.g. animals, musical instruments).	Cards with associated images and sounds (e.g. BS).	Memory, gnosis, attention, and praxis.
4 and 21	*Activity(ies):* Play musical themes related to the authors/performers listed in the roulette. Ask the participant to identify the theme and the respective author/performer; associate each performer with the card with the corresponding image/allusive to the reproduced theme and place it next to the image of the author/performer on the roulette.	Roulette with images of singers associated with the present and the past, cards with images associated with the musical themes performed.	Attention, memory, language, and gnosis.
5 and 22	*Activity(ies):* Give the participant a set of flat geometric figures (e.g. rectangles, circles, squares, triangles) in different colors, sizes, and numbers. Ask the participant to present mathematical operations in which the result corresponds to the number indicated by the therapist and to place the geometrical figures according to the sequence presented through a card given by the therapist.	Flat geometric figures, numbered, with different colours and sizes. Cards with sequences of the geometric figures.	Attention, memory, praxis, calculus, and executive functions.
6 and 23	*Activity(ies):* Give the participant a card with images from the past (e.g. bingo trip to the past). Ask them to identify each of the images and how they differ from the present. Read a story associated with the past (e.g. journey to the past) and ask them to identify the	Bookmarks and a card with images alluding to the past (e.g. BS).	Attention, memory, and language.

	images that are compatible with the narrative. Build a story from an image, tell it in the first person how you lived a certain moment. Elaborate the personal life story.		
7 and 24	*Activity(ies):*<br>Ask the participant to do the cognitive stimulation exercises related to "Attention and Concentration" and "Logical Reasoning".	Cognitive rehabilitation software (e.g, *RehaCom*©).	Attention, memory, calculus, and executive functions.
8 and 25	*Activity(ies):*<br>Give the participant a cardboard with pictures of fruit varieties (e.g. fruit bingo). Ask them to identify each of the fruits on the card and then read an associated story (e.g. the fruit dialogue), identifying the corresponding images.<br>Add new images, associate the fruits with the seasons, build a new story, guess the fruits.	Markers and a card with images alluding to the theme "fruit" (e.g. BS).	Attention, memory, language, and executive functions.
9 and 26	*Activity(ies):*<br>Present the topics of the respective support material (means of transport, household appliances, housing, media, professions, clothing, actors and presenters, politics); ask the participant to mention the content of the images and their relation to the topic in which they are inserted.	Image cards related to the topics identified in the activity (e.g. LPP, pp. 10-27).	Attention, memory, language and executive functions.
10 and 27	*Activity(ies):*<br>Present and explore a topic related to the participant's references and interests (e.g. Portuguese tourist sites), using a digital medium.	Digital presentation (e.g. Power Point presentation, video on the topic to be developed).	Attention, memory, language and gnosis
11 and 28	*Activity(ies):*<br>Place cards with mathematical operations with missing parts on each of the eight parts of the roulette. To the participant: give the individual cards with different numbers to put them into the equation; ask for mathematical operations; order numbers and results; memorize cards and results; simulate purchase operations.	Roulette and cards, on paper, with mathematical operations and results related to the theme "mathematics" (e.g. RM).	Attention, memory, language, calculus and executive functions.
12 and 29	*Activity(ies):*<br>Play music related to each region/district in the country. Ask the participant to identify the respective region using the lyrics of the song and to place a marker in the region/district represented on the country map.	Audio clips of the districts/regions, paper map of the country (e.g. A3 format) and markers.	Attention, memory, language and gnosis
13 and 30	*Activity(ies):*	4 tubes of different colours and small	Attention, memory,

	Give the participant 4 different color tubes (e.g. yellow, red, blue, green). Each tube contains about 30 small sponge cubes painted in the respective colour. Assign a score to each tube (e.g. the value of one unit corresponds to yellow; the value of three units to red; five units to blue; ten units to green). Ask the participant, on the basis of the numbers or mathematical operations indicated by the therapist, to carry out the manipulation of the cubes with a wooden clip.	cubes in the same colours, a wooden clip.	praxis, calculus and executive functions.
14 and 31	*Activity(ies):* Give the participant cards representing amounts of money. Ask the participant to identify the products on a poster. Simulate the purchase of one or more products, so that the participant gives the cards to the therapist for the value of the alleged purchase.	Poster with images of products associated to first necessity products and their respective prices, representative cards of amounts of money.	Attention, memory, calculus and executive functions.
15 and 32	*Activity(ies):* Ask the participant to do cognitive stimulation exercises related to "Spatial Memory" and "Shopping".	Cognitive rehabilitation software (e.g, *RehaCom*©).	Attention, memory, calculus and executive functions.
16 and 33	*Activity(ies):* Provide the participant with a dice with different colors on the faces so that the participant can roll it and, depending on the colour, ask questions about the respective associated theme (each color is assigned a theme, e.g. music, geography of Portugal, gastronomy). Invite and encourage the participant to register and count the correct answers (paper and pencil activity).	Dice with a different colour per side, question cards with the same colour as the dice faces. Each colour is associated with a theme.	Attention, memory, language, praxis and gnosis.
17 and 34	*Activity(ies):* Depending on the topics (e.g., clothing, footwear, food, medicine, technical aids, personal hygiene products, kitchen utensils and parts of the house), ask the participant to group each category into the different parts of the roulette wheel; identify intrusive images; compare images according to certain categories (e.g., weight, value); memorize, remove, and add cards for the participant to identify changes.	Roulette and cards, on paper, with images related to the theme "activities of daily life". (e.g. see the RM material).	Attention, memory, language, praxis, gnosis, calculus and executive functions.
18	Intermediate evaluation	n/a	n/a
35	Post evaluation	n/a	n/a

Abbreviations: RM = *Roletas da Memória*© [Memory Roulettes]; BS = *Bingos Seniores*© [Senior Bingos]; LPP = *Livro do Passado e do Presente*© [Book of the Past and the Present].

Table S3

Baseline scores of the outcomes. Results of between-group comparisons at baseline

	Overall sample (n= 38)	iCS group (n= 19)	Control group (n= 19)		
Baseline assessment	**Mean (SD)**	**Mean (SD)**	**Mean (SD)**	*t*	*p* **value**
**MoCA**	18.84 (3.22)	19.21 (2.80)	18.47 (3.63)	0.701	.488
Range	13-25	15-24	13-25		
**FAB**	10.71 (2.49)	10.74 (2.56)	10.68 (2.50)	0.064	.949
Range	7-17	7-16	8-17		
**IADL**	14.79 (5.32)	15.11 (5.41)	14.47 (5.36)	0.361	.720
Range	8-27	8-27	8-23		
**CES-D**	29.47 (12.25)	28.42 (10.62)	30.53 (13.90)	-0.525	.603
Range	7-53	11-46	7-53		
**WHOQOL-Bref**					
**Physical Health**	50.28 (21.74)	49.44 (21.14)	51.13 (22.87)	-0.237	.814
Range	7.14-100	10.71-78.57	7.14-100		
**Psychological Health**	45.18 (23.42)	44.96 (21.27)	45.39 (25.98)	-0.057	.955
Range	4.17-100	16.67-79.17	4.17-100		
**Social relationships**	37.50 (21.98)	38.16 (21.21)	36.84 (23.29)	0.182	.857
Range	0-75	0-75	0-75		
**Environment**	47.45 (17.30)	46.05 (18.16)	48.85 (16.77)	-0.493	.625
Range	15.63-90.63	15.63-90.63	25-78.13		
**General**	56.91 (20.90)	55.92 (21.40)	57.89 (20.92)	-0.287	.775
Range	12.5-87.5	25-87.5	12.5-87.5		
**SF-36v2**					
**General health**	35.95 (22.58)	37.05 (18.58)	34.84 (26.46)	0.298	.767
Range	0-77	0-67	5-77		
**Physical functioning**	66.97 (33.14)	59.47 (34.88)	74.47 (30.36)	-1.414	.166
Range	0-100	0-100	0-100		
**Role limitations (physical)**	61.51 (25.56)	53.95 (26.45)	69.08 (22.87)	-1.886	.067
Range	12.5-100	12.5-100	18.75-100		
**Bodily pain**	55.45 (27.67)	50.74 (26.76)	60.16 (28.47)	-1.051	.300
Range	10-100	12-100	10-100		
**Role limitations (emotional)**	56.14 (26.75)	49.12 (26.63)	63.16 (25.66)	-1.654	.107
Range	0-100	0-100	25-100		
**Social functioning**	46.05 (26.33)	48.03 (26.77)	44.08 (26.47)	0.457	.650
Range	0-87.5	12.5-87.5	0-87.5		
**Mental health**	40.92 (22.98)	43.68 (20.74)	38.16 (25.29)	0.737	.466
Range	0-85	5-75	0-85		
**Energy/vitality**	31.71 (16.24)	31.05 (15.15)	32.37 (17.67)	-0.246	.807
Range	0-60	0-55	0-60		

Abbreviations: CES-D= Center for Epidemiologic Studies Depression Scale; FAB= Frontal Assessment Battery; IADL= Lawton Instrumental Activities of Daily Living Scale; iCS= Individual Cognitive Stimulation; MoCA= Montreal Cognitive Assessment; SF-36v2= MOS Short Form Health Survey 36 Item v2; WHOQOL-Bref= World Health Organization Quality of Life-Bref.
